# Population Attributable Fraction of Gas Stoves and Childhood Asthma in the United States

**DOI:** 10.3390/ijerph20010075

**Published:** 2022-12-21

**Authors:** Talor Gruenwald, Brady A. Seals, Luke D. Knibbs, H. Dean Hosgood

**Affiliations:** 1 RMI, Carbon-Free Buildings, Boulder, CO 80301, USA; 2 Faculty of Medicine and Health, Sydney School of Public Health, The University of Sydney, Sydney, NSW 2006, Australia; 3 Public Health Unit, Sydney Local Health District, Camperdown, NSW 2050, Australia; 4 Department of Epidemiology and Population Health, Albert Einstein College of Medicine, Bronx, NY 10461, USA

**Keywords:** gas, stove, current, use, asthma, burden, cooking, respiratory, children

## Abstract

Indoor gas stove use for cooking is associated with an increased risk of current asthma among children and is prevalent in 35% of households in the United States (US). The population-level implications of gas cooking are largely unrecognized. We quantified the population attributable fraction (PAF) for gas stove use and current childhood asthma in the US. Effect sizes previously reported by meta-analyses for current asthma (Odds Ratio = 1.34, 95% Confidence Interval (CI) = 1.12–1.57) were utilized in the PAF estimations. The proportion of children (<18 years old) exposed to gas stoves was obtained from the American Housing Survey for the US, and states with available data (n = 9). We found that 12.7% (95% CI = 6.3–19.3%) of current childhood asthma in the US is attributable to gas stove use. The proportion of childhood asthma that could be theoretically prevented if gas stove use was not present (e.g., state-specific PAFs) varied by state (Illinois = 21.1%; California = 20.1%; New York = 18.8%; Massachusetts = 15.4%; Pennsylvania = 13.5%). Our results quantify the US public health burden attributed to gas stove use and childhood asthma. Further research is needed to quantify the burden experienced at the county levels, as well as the impacts of implementing mitigation strategies through intervention studies.

## 1. Introduction

Indoor gas stove use for cooking is associated with an increased risk of current asthma among children [1] and is prevalent in 35% of households in the United States (US), with certain states (e.g., California, Illinois) reaching 68% [2]. Although children’s exposure to gas cooking is widespread, the population-level implications of cooking with gas are largely unrecognized [3]. With this background, we attempted to quantify the population-attributable fraction (PAF) for gas stove use and current childhood asthma in the US, including at the state level, which had never been done. The PAF can be helpful for determining the proportion of preventable disease and may guide public health interventions that aim to reduce disease risk 

## 2. Materials and Methods

First, we sought to update the effect-size estimates by identifying peer-reviewed manuscripts published subsequent to the most recent meta-analysis [1]. We searched PubMed using keywords: “gas cooking and children” OR “gas appliance and children” OR “unvented and children” OR “gas heating and children” OR “gas heater and children”. After including only manuscripts of human studies published in English since 4 January 2013, 357 studies remained for possible inclusion. The title review identified 27 manuscripts as potentially pertinent. Full manuscripts (n = 27) were independently reviewed by co-authors; none reported new associations between gas stove use and childhood asthma specifically in North America or Europe. As a result, effect sizes previously reported for current asthma in North America and Europe combined (weighted by inverse variance; N_studies_ = 10; Odds Ratio (OR) = 1.34, 95% Confidence Interval (CI) = 1.12–1.57) were utilized in the PAF estimations [1]. The combined effect size was based on a North America-specific effect size (N_studies_ = 3; OR = 1.36, 95% CI = 0.76–2.43) and Europe-specific effect size (N_studies_ = 7; OR = 1.34, 95% CI = 1.13–1.60), as reported in a previously published meta-analysis [1]. We combined effect sizes for North America and Europe given the similarities in housing characteristics and gas-stove usage patterns across these geographies. We estimated the proportion of children (<18 years old) exposed to gas stoves in the US and in certain states using the American Housing Survey (AHS) [2]. The AHS is a nationally representative survey of households used to characterize the residential housing stock of the US. Each year, a subset of states is oversampled, allowing for precise estimates of housing characteristics for these states. In the most recent iteration of the survey in 2019, nine states were oversampled, allowing us to estimate PAFs at the national level and for these nine states.

PAFs were estimated using a previously published approach [4]. The approach aimed to quantify our uncertainty regarding the PAF point estimates, given that the inputs to the calculation are based on samples of the population. First, we used the summary statistics of the North America and Europe combined odds ratio described above to generate a distribution of effect sizes. Similarly, we used the summary statistics of the proportion of children exposed to gas cooking to generate distributions of proportions nationally and for each state for which we have data. We then drew a random value from each of these distributions to calculate the PAF according to the following equation:

$$\mathrm{PAF}\;=\frac{p\times \left(RR-1\right)\;}{p\times \left(RR-1\right)+1}$$

where *p* is the proportion of households with children exposed to gas stoves and *RR* is the relative risk of developing asthma given exposure to gas stoves. Note that we used the combined North America and Europe odds ratio for the relative risk in this equation, as childhood asthma in the US remains relatively rare, affecting 1 in 12 children [5]). We repeated this calculation 10,000 times to create distributions of PAFs nationally and for each state, using the same combined odds ratio for national- and state-level calculations We then took the mean of these distributions and calculated their 95% confidence intervals to estimate the PAF of current childhood asthma attributable to gas stove usage. All calculations were performed using the R programming language.

## 3. Results

**Figure 1:**
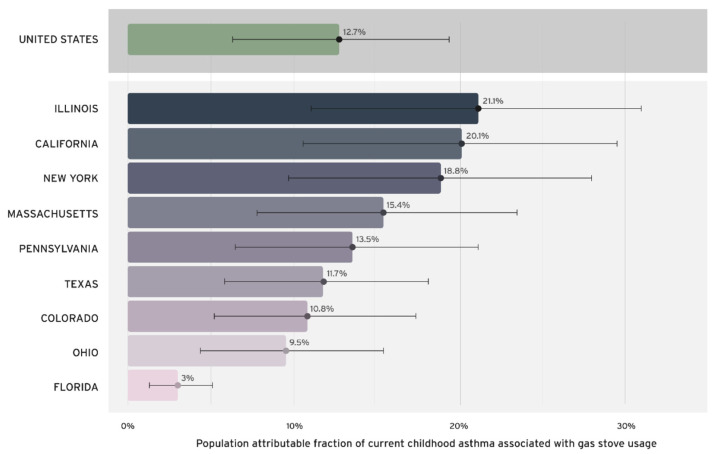
Population attributable fraction (PAF) of current childhood asthma associated with gas-stove use in the United States ^†^. ^†^ PAFs calculated using methods published by Knibbs et al., 2018 [4], with the effect sizes reported by Lin et al., 2013 [1] and the proportion of children (<18 years old) exposed to gas stoves reported by the 2019 American Housing Survey (AHS) [2]. AHS only included information on gas stove use for the nine states included. No data were available for other states.

We found that 12.7% (95% CI = 6.3–19.3%) of current childhood asthma in the US is attributable to gas stove use (Figure 1). At the state level, the proportion of childhood asthma that could be theoretically prevented if gas stove use was not present (e.g., state-specific PAFs) varied. Illinois experiences the highest burden (21.1%), followed by California (20.1%), New York (18.8%), Massachusetts (15.4%), and Pennsylvania (13.5%). Texas, Colorado, and Ohio all experience burdens around 10%. Florida experiences the lowest burden (3%). The state-level PAFs differ due to varying exposure to gas stoves among children. In Illinois, for example, approximately 79.1% of households with children cook with gas, whereas in Florida, the figure is only 9.1%. States with a higher percentage of children living in households with gas stoves have higher proportions of current childhood asthma attributable to gas stove usage.

## 4. Discussion

Our results quantify the US public health burden attributed to gas-stove use and childhood asthma. Our study strengths include utilization of peer-reviewed effect sizes [1] and existing PAF models [4]. While we are the first to report these PAFs at national and state-specific levels using nationally representative AHS data, we were limited by the number of states (n = 9) with available data. Nonetheless, our results align with a cross-sectional study which found that the use of a gas stove or oven for heat was a main risk factor for doctor-diagnosed asthma in US children under age six [6].

We note that PAF analyses have limitations. For example, we rely on aggregate data, although we have quantified our uncertainty and have still found a significant public health burden. Additionally, our interpretation of the PAF as the proportion of preventable disease rests on the assumptions that (1) exposure to gas cooking among children is orthogonal to other risk factors such as exposure to tobacco smoke and (2) that we can conceive of a broad-based public health intervention to reduce the disease risk in children exposed to gas cooking to that of the unexposed, while holding all other risk factors equal. 

We posit that there are two such interventions to reduce the childhood asthma disease risk attributable to gas stoves: (1) removing the source by replacing gas cooking with cleaner alternatives (e.g., electric), and (2) reducing exposure through source ventilation (e.g., range hoods). Notably, ventilation is associated with the reduction, but not elimination, of childhood asthma risk [7]. Moreover, high-efficiency range hoods are not practical in all settings (e.g., apartment buildings). In homes with range hoods, they may not vent outdoors or be effective at removing combustion pollutants, and residents simply may not use them [8]. Indeed, according to National Health and Nutrition Examination Survey data, among children living in households that use gas stoves, only 21.1% live in households where the stove’s exhaust vent is always used.

## 5. Conclusions

In conclusion, 12.7% of current childhood asthma nationwide is attributed to gas stove use, which is similar to the childhood asthma burden attributed to secondhand smoke exposure [9]. Gas stove usage should be considered in multi-faceted asthma prevention approaches. Given that this exposure is preventable, our study demonstrates that known mitigation strategies will lessen childhood asthma burden from gas stoves, particularly in states with elevated PAFs. Further research is needed to quantify the burden experienced at the state and county levels, as well as the impacts of implementing mitigation strategies through intervention studies.

## Data Availability

All data are publicly available as described in the Section 2.
